# MIDLIFE HEALTH FACTORS AND LATER-LIFE COGNITIVE DISEASE: RESULTS FROM THE 1998-2014 HEALTH AND RETIREMENT STUDY

**DOI:** 10.1093/geroni/igz038.3451

**Published:** 2019-11-08

**Authors:** Benson Wu, Mohammad Usama Toseef, Wassim Tarraf, Hector M González

**Affiliations:** 1 Department of Neurosciences at UC San Diego School of Medicine, La Jolla, California, United States; 2 Wayne State University, Detroit, Michigan, United States; 3 University of California, San Diego, San Diego, California, United States

## Abstract

Understanding lifecourse determinants of older-age health outcomes is indispensable for resources planning and optimizing public health in light of continued gains in longevity in the US and worldwide. Data increasingly points to midlife health and modifiable risk factors as critical targets for improving older-age health outcomes and mitigating potential cognitive impairment and disease. We used 16-years of biennial data (1998-2014) from the Health and Retirement Study (unweighted-n=6,724), to examine how a comprehensive battery of midlife (age 50-64 years) health measures (disability, physical function, comorbid conditions, and self-reported health) affect cognitive status (using Langa-Weir criteria: Normal, Cognitively Impaired Not Dementia (CIND), and Dementia) and death 16-years later. Additionally, we test for racial/ethnic and gender modifications in the effects of these conditions on the outcomes of interest. We used survey multinomial logistic regression models adjusting for predisposing sociodemographic factors, health-enabling economic characteristics and health behaviors. Relative risk ratios (RRR) across all unadjusted models varied from 1.36-4.84 and 1.36-3.31 for those with dementia and who died in 2014 respectively, suggesting worse health outcomes in midlife are associated with higher dementia/mortality risk in later-life. After covariates-adjustment, comorbidities (RRR=1.15[1.04,1.27]) and Self-reported Health (RRR=1.36[1.22,1.52]) were associated with CIND, and attenuation was particularly pronounced for IADLS (RRR=3.15[2.25,4.43]) and Fine Motor Skills (RRR=1.94[1.46,2.57]) for individuals with dementia in 2014. Neither sex nor race/ethnicity modified these associations. Modifying the midlife health profile of US adults can yield important public health savings and reductions in structural and social health burdens through extenuating the prevalence of dementias and reducing excess mortality.

